# Validation of the Performance of International Ovarian Tumor Analysis (IOTA) Methods in the Diagnosis of Early Stage Ovarian Cancer in a Non-Screening Population

**DOI:** 10.3390/diagnostics7020032

**Published:** 2017-06-02

**Authors:** Wouter Froyman, Laure Wynants, Chiara Landolfo, Tom Bourne, Lil Valentin, Antonia Testa, Povilas Sladkevicius, Dorella Franchi, Daniela Fischerova, Luca Savelli, Ben Van Calster, Dirk Timmerman

**Affiliations:** 1 Department of Development and Regeneration, KU Leuven, Leuven post code3000, Belgium; wouter.froyman@uzleuven.be (W.F.); laure.wynants@kuleuven.be (L.W.); chiara.landolfo@kuleuven.be (C.L.); t.bourne@imperial.ac.uk (T.B.); ben.vancalster@kuleuven.be (B.V.C.); 2 Department of Obstetrics and Gynecology, University Hospitals Leuven, Leuven 3000, Belgium; 3 Queen Charlotte’s and Chelsea Hospital, Imperial College, London W12 0HS, UK; 4 Department of Obstetrics and Gynecology, Skåne University Hospital Malmö, Lund University, Malmö 20502, Sweden; lil.valentin@med.lu.se (L.V.); povilas.sladkevicius@med.lu.se (P.S.); 5 Department of Oncology, Catholic University of the Sacred Heart, Rome 00168, Italy; atesta@rm.unicatt.it; 6 Preventive Gynecology Unit, Division of Gynecology, European Institute of Oncology, Milan 20141, Italy; dorella.franchi@ieo.it; 7 Gynecological Oncology Center, Department of Obstetrics and Gynecology, Charles University, Prague 12108, Czech Republic; Daniela.Fischerova@seznam.cz; 8 Department of Obstetrics and Gynecology, S. Orsola-Malpighi Hospital, University of Bologna, Bologna 40138, Italy; luca.savelli@aosp.bo.it

**Keywords:** ovary, ovarian neoplasms, early detection of cancer, diagnostic imaging, ultrasonography, risk assessment, logistic models

## Abstract

Background: The aim of this study was to assess and compare the performance of different ultrasound-based International Ovarian Tumor Analysis (IOTA) strategies and subjective assessment for the diagnosis of early stage ovarian malignancy. Methods: This is a secondary analysis of a prospective multicenter cross-sectional diagnostic accuracy study that included 1653 patients recruited at 18 centers from 2009 to 2012. All patients underwent standardized transvaginal ultrasonography by experienced ultrasound investigators. We assessed test performance of the IOTA Simple Rules (SRs), Simple Rules Risk (SRR), the Assessment of Different NEoplasias in the adneXa (ADNEX) model and subjective assessment to discriminate between stage I-II ovarian cancer and benign disease. Reference standard was histology after surgery. Results: 230 (13.9%) patients proved to have stage I–II primary invasive ovarian malignancy, and 1423 (86.1%) had benign disease. Sensitivity and specificity with respect to malignancy (95% confidence intervals) of the original SRs (classifying all inconclusive cases as malignant) were 94.3% (90.6% to 96.7%) and 73.4% (71.0% to 75.6%). Subjective assessment had a sensitivity and specificity of 90.0% (85.4% to 93.2%) and 86.7% (84.9% to 88.4%), respectively. The areas under the receiver operator characteristic curves of SRR and ADNEX were 0.917 (0.902 to 0.933) and 0.905 (0.920 to 0.934), respectively. At a 1% risk cut-off, sensitivity and specificity for SRR were 100% (98.4% to 100%) and 38.0% (35.5% to 40.6%), and for ADNEX were 100% (98.4% to 100%) and 19.4% (17.4% to 21.5%). At a 30% risk cut-off, sensitivity and specificity for SRR were 88.3% (83.5% to 91.8%) and 81.1% (79% to 83%), and for ADNEX were 84.5% (80.5% to 89.6%) and 84.5% (82.6% to 86.3%). Conclusion: This study shows that all three IOTA strategies have good ability to discriminate between stage I-II ovarian malignancy and benign disease.

## 1. Introduction

Ovarian tumors are common in women of all ages [1,2,3]. It has been estimated that in the female population, the lifetime risk of undergoing surgery for a suspected ovarian neoplasm is 5–10% [4]. However, the incidence of ovarian cancer is low. In Europe, there were 65,538 new cases during 2012, with an age-adjusted incidence rate of 13.1 per 100,000 women. Still, ovarian cancer is an important health problem in gynecology, as it is the most lethal gynecological malignancy, with 42,700 deaths occurring in 2012 in Europe (mortality rate 7.6 per 100,000) [5]. This accounts for 5% of all cancer deaths in women, which makes ovarian cancer the sixth most lethal cancer in females in Europe [6].

In recent decades, despite advances in cytoreductive radical surgery and cytotoxic chemotherapy, we have seen only a marginal improvement in the overall survival of patients with ovarian cancer [7].

Almost 60% of patients are diagnosed with advanced disease with regional or distant spread and an unfavorable long-term prognosis. Five-year relative survival is 46% for all International Federation of Gynecology and Obstetrics (FIGO) stages [8], but ranges from 90% at Stage I to 4% for Stage IV disease [6,8]. Therefore, attention for the development of strategies to detect ovarian malignancy at an early stage using imaging and/or biomarkers is increasing, in order to improve patient survival. This idea is reflected in the conduction of several large ovarian cancer screening trials [9,10,11], but also plays an important role in clinical management of the non-screening population.

Early detection of cancer means that treatment is not delayed and that appropriate staging can be carried out in specialized surgical centers, which is known to improve survival [12,13,14,15].

The best ultrasound method for discrimination between benign and malignant adnexal masses is the subjective assessment of ultrasound findings by an experienced ultrasound examiner [16,17,18]. However, as such expert knowledge is not available in each center, the International Ovarian Tumor Analysis (IOTA) study aims to develop diagnostic algorithms to assist clinicians in characterizing adnexal pathology, irrespective of their level of expertise. The IOTA group initially published a consensus paper in order to standardize terms, definitions, and measurements used to assess ovarian pathology [19]. By prospectively investigating patients presenting with an adnexal mass (i.e., non-screening population), this formed the basis for the development of different IOTA methods such as the Simple Rules (SRs), which are based on five ultrasound features suggestive for a benign lesion (B-features) and five features suggestive for a malignant lesion (M-features) [20]. The IOTA SRs have become very popular because they are easy to use, without the need for any calculation. They have been extensively validated and are incorporated in international guidelines [21,22]. Two systematic reviews and meta-analyses have concluded that the IOTA SRs are one of the best performing available diagnostic methods for differentiating between benign and malignant adnexal masses [18,23]. Shortcomings of the SRs are that there are inconclusive results in a proportion of cases (when B and M features apply or when no features apply) and the absence of an estimated risk of malignancy. Therefore, the ultrasound features used in the SRs have recently been used to calculate a risk of malignancy, leading to the Simple Rules Risk (SRR) model [24]. Another logistic regression model developed by the IOTA group is the Assessment of Different NEoplasias in the adneXa (ADNEX) model. As a multiclass prediction model, ADNEX not only calculates the likelihood of malignancy in adnexal masses, but also divides this into the likelihood that the mass is borderline malignant, stage I primary invasive ovarian cancer, stage II–IV primary invasive ovarian cancer, or a metastasis in the ovary from another primary tumor [25]. The performance of ADNEX is at least as good as the performance of previous IOTA methods, as confirmed by external validation studies [26,27,28,29,30]. The ADNEX model is available online and in mobile applications (www.iotagroup.org/adnexmodel/).

Given the good performance of IOTA strategies in discriminating between benign and malignant disease in patients presenting with an adnexal mass prior to surgery, we are often confronted with the question on how IOTA methods could potentially improve detection in ovarian cancer screening. For the purpose of this special issue of Diagnostics, we assessed and compared the test performance of various diagnostic IOTA methods and subjective assessment to identify early stage, i.e., FIGO stage I and II [8], primary invasive ovarian malignancy in a non-screening population.

## 2. Materials and Methods

### 2.1. Patients

This study was performed on data of IOTA phase 3 [31], a multicenter cross-sectional diagnostic accuracy study with prospective data collection. Patients were recruited in 18 centers in six countries (Sweden, Belgium, Italy, Poland, Spain, and Czech Republic) between October 2009 and May 2012. The participating centers were either oncology referral centers (i.e., tertiary centers with a specific gynecological oncology unit) or general hospitals and units with a special interest in gynecological ultrasound. Ethics approval for IOTA 3 was obtained by the ethics committee of the University Hospitals Leuven (B32220095331/S51375 approved 21 January 2009) as the main investigating center as well of the local ethics committees of all contributing centers.

Patients were eligible for IOTA 3 if they presented with at least one adnexal mass (ovarian, para-ovarian, or tubal), underwent standardized transvaginal ultrasonography by a principal investigator at one of the participating centers, and were then selected for surgical intervention by the managing clinician. All examiners were experienced in gynecologic ultrasound. Details on the ultrasound examination technique and the IOTA terms and definitions used to describe adnexal pathology have been published elsewhere [19]. More information on data collection can be found in the original IOTA 3 publication [31]. The pathologist was blinded to the predicted outcomes of the index tests being compared.

For the purpose this study, only patients having a histopathology diagnosis of a benign mass or FIGO stage I and II [8] invasive (epithelial or non-epithelial) ovarian malignancy were considered for analysis.

### 2.2. Diagnostic Models

Three diagnostic IOTA methods for the assessment of adnexal masses (the original SRs, SRR and ADNEX) were evaluated in terms of their ability to discriminate between benign disease and stage I–II primary ovarian malignancy. These methods were developed on data of earlier IOTA phases. Hence, this is a temporal validation study, including new centers. The original IOTA SRs result in a classification of ovarian masses as benign, malignant, or inconclusive. In this work, we classified inconclusive cases as malignant. The SRR yields a predicted probability of ovarian malignancy. The ADNEX model provides the predicted risks of four different subclasses of malignant adnexal tumors (borderline, stage I invasive, stage II-IV invasive or metastatic cancer). When using the ADNEX model, the probability of malignancy is computed as the sum of the predicted probabilities for all malignant subtypes (including borderline tumors). We validated the version of ADNEX that does not use serum cancer antigen 125 (CA125) measurements as a predictor, because CA125 results are not always available in women with benign or stage I–II tumors (results for serum CA125 measurements were missing for 45% of women in our database). ADNEX with and without CA125 has similar ability to predict malignancy [25]. Both SRR and ADNEX were initially developed on data from IOTA phases 1 and 2, validated on data from IOTA 3, and then refitted on all data [24,25]. In this study, we used the initial versions of SRR and ADNEX that were not refitted using IOTA 3 data. We also evaluated the performance of subjective assessment.

### 2.3. Statistical Methods

All strategies were evaluated in terms of their ability to discriminate between benign and malignant masses. The area under the receiver-operator characteristic curve (AUC) was computed for ADNEX and the SRR. We also calculated the sensitivity and specificity for ADNEX and the SRR at risk thresholds of 1%, 10%, 20%, and 30%, as well the sensitivity and specificity of the original SRs (classifying inconclusive results as malignant) and subjective assessment. Subgroup analyses were performed for pre- and postmenopausal women. R software (version 3.3.1.) was used for all calculations (R: A language and environment for statistical computing. R Foundation for Statistical Computing, Vienna, Austria, Available online: http/www.r-project.org/). The pROC package and binom packages were used to calculate Delong [32] and Wilson [33] confidence intervals for AUCs and sensitivity/specificity, respectively. The Transparent Reporting of a multivariable prediction model for Individual Prognosis or Diagnosis (TRIPOD) guidelines [34] were used for reporting in this study.

## 3. Results

In total, 2541 women with adnexal masses were enrolled in IOTA phase 3. We excluded 138 women from the final data set after the application of exclusion criteria [31].

Of the remaining 2403 patients, 1423 had a benign mass. Patients with borderline tumors, stage III–IV primary invasive malignancies, and metastatic cancer were excluded from the analysis. The resulting database for analysis consisted of 1653 women from 18 centers, 230 of which had stage I–II invasive ovarian malignancy. Patient and tumor characteristics are represented in Table 1. Of the women included, 34.6% were postmenopausal. Histology findings are listed in Table 2.

Results are shown as medians (interquartile range) for continuous and ordinal variables, and as *N* (%) for categorical variables. Possible values for “Number of locules” are 1 = presence of one locule, 2 = presence of two locules, 3 = presence of three locules, 4 = presence of four locules, 5 = presence of five to ten locules, 6 = presence of more than ten locules. Possible values for “Number of papillary structures” are 1 = presence of one papillary structure, 2 = presence of two papillary structures, 3 = presence of three papillary structures, 4 = presence of more than three papillary structures.

Regarding the identification of stage I–II primary ovarian malignancy as malignant disease, the original SRs (classifying inconclusive cases as malignant) had a sensitivity and specificity (95% confidence intervals) of 94.3% (90.6% to 96.7%) and 73.4% (71.0% to 75.6%). Subjective assessment had a sensitivity and specificity of 90.0% (85.4% to 93.2%) and 86.7% (84.9% to 88.4%), respectively. Considering the discrimination of benign and malignant disease in the study population of patients with benign masses and stage I–II primary ovarian malignancy the AUCs of the SRR and ADNEX model were 0.917 (0.902 to 0.933) and 0.920 (0.905 to 0.934), respectively. The sensitivity and specificity for these risk prediction models differ depending on the selected risk threshold to predict malignancy. Table 3 summarizes the sensitivity and specificity for the two models at different risk thresholds.

When stratifying for menopausal status, the original SRs (classifying inconclusive cases as malignant) had a sensitivity and specificity of 94.3% (88.1% to 97.4%) and 77.3% (74.5% to 79.8%) in premenopausal patients, and 94.4% (88.9% to 97.3%) and 64.9% (60.3% to 69.2%) in postmenopausal patients. Subjective assessment had a sensitivity and specificity of 87.6% (80.0% to 92.6%) and 89.0% (86.9% to 90.8%) in premenopausal patients, and 92.0% (85.9% to 95.6%) and 81.7% (77.8% to 85.0%) in postmenopausal patients.

In premenopausal women, the AUCs of the SRR and ADNEX model were 0.932 (0.913 to 0.950) and 0.932 (0.913 to 0.950), respectively. In postmenopausal women, the AUCs of the SRR and ADNEX model were 0.882 (0.853 to 0.912) and 0.885 (0.858 to 0.912), respectively.

Table 4 summarizes the sensitivity and specificity for the two models at different risk thresholds for pre- and postmenopausal women.

## 4. Discussion

This validation of IOTA ultrasound-based rules and risk prediction models showed good test performance to discriminate between benign disease and stage I–II ovarian malignancy before surgery.

The strength of this study is the use of a large international database in which information was prospectively collected using well-defined terms, definitions, and measurement methods [19]. The large sample size and the participation of different types of centers are likely to yield generalizable results.

A limitation of our study is that the diagnostic methods were validated exclusively on patients who underwent surgery. This does not reflect clinical practice, where some masses are managed expectantly, but it allowed us to use histological diagnosis as the gold standard. We are awaiting the results of IOTA phase 5, in which IOTA methods are validated on consecutively collected adnexal masses of all kinds, including those managed conservatively. A second limitation is that all ultrasound examiners in the study were very experienced. Our results might not necessarily be applicable to less experienced operators. However, published studies have shown that the IOTA SRs and ADNEX retain their performance in the hands of less experienced examiners [27,28,35,36,37,38,39,40,41]. This is likely to be true also for the SRR model, because the same ultrasound variables are used in the original SRs are used to calculate the risks of the SRR model. A third limitation of our study is that not all histopathology information necessary to classify the tumors into type I and type II epithelial malignancies had been collected. This is explained by the fact that patient recruitment for IOTA 3 started in 2009, before the dualistic model of ovarian carcinogenesis [42] was widely accepted.

The findings of our study show that the performance of IOTA methods for differentiating benign disease from stage I–II primary ovarian malignancy is not much lower than the performance for the discrimination of benign from all malignant disease (all malignant subtypes grouped together) [24,25,31]. In the original publications including all IOTA 3 patients, validation AUCs (95% confidence intervals) regarding discrimination between benign and malignant disease for SRR and ADNEX (without CA125) were 0.917 (0.902–0.930) [24] and 0.932 (0.922–0.941) [25], respectively. Sensitivity and specificity for the original SRs on validation in the same population were 95.3% (93.1% to 96.9%) and 74.1% (67.7% to 79.7%), respectively [24,25,31].

Borderline malignant tumors were excluded from our analysis. These tumors are known to be more difficult to classify as benign or malignant [25,43,44]. On the other hand, borderline (i.e., non-invasive malignant) ovarian tumors rarely precede invasive epithelial ovarian carcinoma [45,46]. More clinically relevant is the correct identification of early stage primary invasive tumors, where prompt and adequate surgical staging is important for improving survival [47]. Detection of stage I-II ovarian cancer is particularly important for screening for ovarian cancer to be successful. The aim of screening for ovarian cancer is to decrease ovarian cancer mortality. For this to be possible, screening should result in a shift towards earlier stages at detection, i.e., the detection rate of stage I–II ovarian cancer should be high. However, a shift towards earlier detection of ovarian cancer has been shown in only two [9,11] of three randomized controlled trials [9,10,11] on ovarian cancer screening, and none of the two completed screening trials has shown conclusive evidence of decreased ovarian cancer mortality in the screened group [10,11]. In the two completed randomized trials on ovarian cancer screening [10,11], the ultrasound criteria to define an abnormal screening result were subjective or arbitrary. As a result, many patients with benign disease were scheduled for surgery, i.e., a large number of operations were performed to detect one cancer case. We speculate that the positive predictive value of an abnormal screen result could be improved if the IOTA methods were used to define an abnormal scan result. To the best of our knowledge, the discriminative or predictive performance of the IOTA methods has never been assessed in a screening population.

About 90% of invasive malignant ovarian tumors are epithelial [48]. The dualistic model proposed by Shih and Kurman highlights the heterogeneity of ovarian carcinoma and implies that ultrasound-based screening will not be effective in detecting all types of ovarian carcinoma. Type I tumors (low-grade serous, low-grade endometrioid, clear cell, and mucinous) are slow growing, attain a large size while still confined to the ovary, and are thus likely to be detected early by transvaginal ultrasound. Unfortunately, these lesions constitute only 25% of ovarian cancers and account for only approximately 10% of ovarian cancer deaths. On the other hand, type II tumors (high-grade serous and undifferentiated carcinomas, and malignant mixed mesodermal tumors (carcinosarcomas)) represent 75% of all ovarian carcinomas, are responsible for 90% of ovarian cancer deaths, and may originate outside the ovary. These tumors are almost never confined to the ovary at diagnosis, making their diagnosis at an early point in the disease course challenging [42,49]. To allow detection of this aggressive type of ovarian cancer, there is ongoing search for sensitive biomarkers expressed early in ovarian carcinogenesis. More recently, there is increasing interest in the use of genomic profiling as a potential candidate for the detection of ovarian malignancies [50,51]. Further research should explore whether IOTA methods may serve as a second stage test in a program of ovarian cancer screening to avoid unnecessary surgery without delaying a diagnosis of ovarian cancer.

## 5. Conclusions

This analysis shows that the IOTA methods have good ability to discriminate between stage I–II ovarian malignancy and benign adnexal lesions prior to surgery. The potential use of IOTA methods as a second stage test in ovarian cancer screening should be the subject of further investigation.

## Figures and Tables

**Table 1:** Descriptive statistics of the sample.

Variable	Result	
	Benign Tumor	Early Stage Malignancy (I and II)
*N*	1423 (86.1%)	230 (13.9%)
Age (years)	44 (33 to 56)	55 (42 to 66)
Postmenopausal	447 (31.4%)	125 (54.3%)
CA125 (IU/L), if available ^a^	21 (12 to 46)	55 (20 to 207)
Maximum tumor diameter (mm)	64 (47 to 90)	103 (68 to 143)
Presence of solid components	474 (33.3%)	214 (93.0%)
Maximum diameter of the solid component (if any, mm)	28 (13 to 54)	62 (37 to 93)
Locularity		
Unilocular	595 (41.8%)	2 (0.9%)
Unilocular-solid	141 (9.9%)	34 (14.8%)
Multilocular	354 (24.9%)	14 (6.1%)
Multilocular-solid	179 (12.6%)	93 (40.4%)
Solid	154 (10.8%)	87 (37.8%)
Number of locules (if any)	1 (1 to 3)	5 (1.5 to 6)
Acoustic shadows	265 (18.6%)	17 (7.4%)
Intratumoral blood flow		
No blood flow	574 (40.3%)	5 (2.2%)
Minimal blood flow	563 (39.6%)	54 (23.5%)
Moderate blood flow	239 (16.8%)	95 (41.3%)
Very strong blood flow	47 (3.3%)	76 (33.0%)
Irregular internal cyst wall	385 (27.1%)	151 (65.7%)
Presence of ascites	18 (1.3%)	33 (14.3%)
Presence of papillary structures	180 (12.6%)	54 (23.5%)
Number of papillary structures (if present)	1 (1 to 3)	3 (2 to 4)

^a^ CA125: cancer antigen 125. There were 683 (48%) missing values for CA125 for benign tumors and 64 (28%) for early stage tumors.

**Table 2:** Overview of histologic outcomes (*N*, %).

Histology	*N* (%)
Endometrioma	344 (20.8%)
Teratoma	231 (14.0%)
Simple cyst + parasalpingeal cyst	106 (6.4%)
Functional cyst	40 (2.4%)
Hydrosalpinx + salpingitis	47 (2.8%)
Peritoneal pseudocyst	18 (1.1%)
Abscess	17 (1.0%)
Fibroma	130 (7.9%)
Serous cystadenoma	259 (15.7%)
Mucinous cystadenoma	183 (11.1%)
Rare benign	48 (2.9%)
Primary invasive (epithelial) cancer stage I	128 (7.7%)
Primary invasive (epithelial) cancer stage II	47 (2.8%)
Rare primary invasive malignancy stage I or II *	55 (3.3%)

* Includes germ cell tumors and sex cord-stromal tumors.

**Table 3:** Sensitivity and specificity for Assessment of Different NEoplasias in the adneXa (ADNEX) model and Simple Rules Risk (SRR) model at various risk thresholds (percent (95% confidence interval)).

Risk Threshold	Statistic	ADNEX	SRR
1%	Sensitivity	100.0%	100.0%
		(98.4% to 100.0%)	(98.4% to 100.0%)
	Specificity	19.4%	38.0%
		(17.4% to 21.5%)	(35.5% to 40.6%)
10%	Sensitivity	97.4%	97.0%
		(94.4% to 98.8%)	(93.9% to 98.5%)
	Specificity	69.5%	65.1%
		(67.1% to 71.8%)	(62.6% to 67.6%)
20%	Sensitivity	91.3%	94.3%
		(87.0% to 94.3%)	(90.6% to 96.7%)
	Specificity	79.7%	74.3%
		(77.5% to 81.7%)	(71.9% to 76.5%)
30%	Sensitivity	84.5%	88.3%
		(80.5% to 89.6%)	(83.5% to 91.8%)
	Specificity	84.5%	81.1%
		(82.6% to 86.3%)	(79.0% to 83.0%)

**Table 4:** Sensitivity and specificity by menopausal status for Assessment of Different NEoplasias in the adneXa (ADNEX) model and Simple Rules Risk (SRR) model at various risk thresholds (percent (95% confidence interval)).

Risk Threshold	Statistic	ADNEX		SRR	
		Premenopausal	Postmenopausal	Premenopausal	Postmenopausal
1%	Sens	100.0%	100.0%	100.0%	100%
		(96.5% to 100.0%)	(97.0% to 100%)	(96.5% to 100.0%)	(97.0% to 100.0%)
	Spec	25.8%	5.4%	41.4%	30.6%
		(23.2% to 28.7%)	(3.6% to 7.9%)	(38.3% to 44.5%)	(26.6% to 35.1%)
10%	Sens	94.3%	100%	98.1%	96.0%
		(88.1% to 97.4%)	(97.0% to 100%)	(93.3% to 99.5%)	(91.0% to 98.3%)
	Spec	77.8%	51.5%	70.6%	53.2%
		(75.1% to 80.3%)	(46.8% to 56.1%)	(67.7% to 73.4%)	(48.6% to 57.8%)
20%	Sens	86.7%	95.2%	94.3%	94.4%
		(78.9% to 91.9%)	(89.9% to 97.8%)	(88.1% to 97.4%)	(88.9% to 97.3%)
	Spec	85.6%	66.9%	78.5%	65.1%
		(83.2% to 87.6%)	(62.4% to 71.1%)	(75.8% to 80.9%)	(60.6% to 69.4%)
30%	Sens	78.1%	92.0%	84.8%	91.2%
		(69.3% to 84.9%)	(85.9% to 95.6%)	(76.7% to 90.4%)	(84.9% to 95.0%)
	Spec	89.5%	73.6%	84.1%	74.5%
		(87.5% to 81.3%)	(96.3% to 77.5%)	(81.7% to 86.3%)	(70.3% to 78.3%)
